# Pattern of antibiotic use for acute respiratory infections among out-patients in South Asian Region: Protocol for a systematic review

**DOI:** 10.1097/MD.0000000000022398

**Published:** 2021-01-29

**Authors:** Saif Al-Amin, Md Zakiul Hassan, K.M. Saif-Ur-Rahman, Muhammad Abdul Baker Chowdhury, Sharon D. Morrison, Sara B. Donevant, Fahmida Chowdhury

**Affiliations:** a Department of Public Health Education, University of North Carolina at Greensboro, Greensboro, NC; b Programme for Emerging Infections, Infectious Diseases Division; c Health Systems and Population Studies Division, ICDDRB, Dhaka, Bangladesh; d Department of Public Health and Health Systems, Graduate School of Medicine, Nagoya University, Nagoya, Japan; e Department of Emergency Medicine, University of Florida College of Medicine, Gainesville, FL; f College of Nursing, University of South Carolina, Columbia, SC; g Nuffield Department of Medicine, University of Oxford.

**Keywords:** acute respiratory infection, antibiotics, antimicrobial resistance, out-patients, self-medication, South Asia

## Abstract

**Background:**

South Asian region has been experiencing the increasing burden of antimicrobial resistance (AMR) primarily due to over and irrational prescribing of antibiotics. Acute respiratory infections (ARIs) are the leading cause of out-patients’ visits in the region. Despite commonly known viral aetiology, ARI is the single largest reason for antibiotic prescriptions contributing the exponential growth of AMR in the region. Collated data on antibiotic consumption for ARI at outpatients and resistance pattern of respiratory pathogen are lacking in the region.

**Methods:**

MEDLINE, Cochrane, CINAHL Plus (EBSCO), and Web of Science will be searched for eligible papers. Titles and abstracts, and full texts of the relevant studies will be screened by 2 independent reviewers against the inclusion criteria. Data extraction and quality of the studies will be assessed by 2 reviewers independently using the JBI Critical Appraisal Tools. A third reviewer will resolve any disagreement at any point between 2 reviewers.

**Results:**

The review will assess proportions of ARI patients receiving antibiotic therapy and types of antibiotics prescribed among outpatients of all ages in South Asia. This review will also assess the pattern of antimicrobial resistance among respiratory pathogens causing ARI in the region.

**Conclusions:**

This systematic review will evaluate published literature, summarize the existing data on the antibiotic prescribing patterns for outpatients with ARI in South Asia. The holistic finding of the proportion of patients receiving antibiotic therapy for ARI, proportion of different types of antibiotic received, and resistance against respiratory pathogen might guide future research. This underscores a need for formulating regional and national policy for AMR mitigation strategy, and revising clinical practice guidelines for the clinician to ensure rational use of antibiotics for ARI.

**PROSPERO:**

registration no: CRD42018116658

## 1 Introduction

Antimicrobial resistance (AMR) is a growing global public health concern. Irrational use of antibiotics has repeatedly been linked with the rapid growth of AMR globally. Antibiotic consumption has been increased significantly over the last decades, most prominently in low and middle-income countries (LMIC).^[1]^ Factors leading to increased antibiotic consumption in LMIC include increased morbidity and mortality due to illness, significant patient volumes, lack of diagnostic facility, and attitudes of both patients and health care providers.^[2]^ In South Asia, the pervasive use of antibiotics is also due to weak drug regulatory regime.^[3]^ In this region, antibiotics are widely available as over-the-counter (OTC) drugs and such easy availability has triggered indiscriminate antibiotic prescription equally by formal and informal sector.^[4–10]^

Acute respiratory infections (ARIs) are one of the primary causes of morbidity and mortality in developing countries.^[11]^ In 2010, the World Health Organization (WHO) reported 1.9 to 2.2 million ARI deaths among children globally. About 70% of these deaths occurred in Africa and Southeast Asia.^[12]^ It is estimated that 20% to 40% of outpatient consultations and 12% to 35% of pediatric admissions are due to ARI.^[13]^ Most ARIs are viral in origin and clinical guidelines do not recommend routine use of antibiotic for ARI. Despite evidence of no benefit, ARI has been the leading cause of antibiotic prescription at outpatient with over 80% receiving antibiotic unnecessarily in LMICs.^[14]^

Such unrestricted use of antibiotics propelling the growth of AMR. A prospective surveillance study conducted in 11 Asian countries during 2008 to 2009 among the patients with pneumococcal pneumonia revealed that resistance to erythromycin was highly prevalent (72%) and multidrug resistance (MDR) was reported for 59% of *Streptococcus pneumoniae* isolates.^[15]^ Different studies conducted in South Asian countries have also reported a similar pattern of antibiotic resistance among respiratory pathogens.^[16–19]^ However, most of these studies on AMR and prescribing patterns are reported in India and a few studies have been reported the situation of Bangladesh, Sri Lanka, Nepal, and Pakistan. Other countries of the region such as Bhutan, Nepal, Maldives, and Afghanistan have limited data to study on. The scarcity of South Asian regional data on AMR against respiratory pathogen and the antibiotic prescription pattern in ARI among the outpatients could be considered as a potential research gap and requires further studies. The purpose of this systematic review is to provide a summary of the existing data on the proportion of patients receiving antibiotic for an ARI episode, including types of antibiotics prescribed, prevalence, and pattern of antibiotic resistance against respiratory pathogens. This systematic review will provide holistic data on antibiotic use and resistance patterns in the South Asian region, which might guide policy decisions regarding promoting rational use of antibiotics in the region. Furthermore, these findings may also support clinicians with a better understanding of the current antimicrobial resistance pattern in the region and more informed choices of antibiotic during prescribing.

## 2 Review question

(1) What proportions of patients with an episode of ARI Receive antibiotic therapy including types of antibiotics at Outpatient facilities in South Asia?
(2) What is the pattern of antimicrobial resistance among respiratory pathogens causing ARI in South Asia?

## 3 Methods

The systematic review will be conducted in accordance with the Preferred Reporting Items for Systematic Review and Meta-Analyses Protocols (PRISMA-P).^[20]^ The review was registered in PROSPERO with registration number of CRD42018116658.

### 3.1 Inclusion and exclusion criteria

Studies will be selected according to the criteria outlined. All the inclusion and exclusion criteria are summarized in Table 1.

**Table 1:** Inclusion and exclusion criteria.

Inclusion	Exclusion
Cross-sectional studies, Case–control studies, Cohort studies. Randomized control trials (RCTs)	Case study, Case series
Studies conducted within 8 South Asian Countries (Afghanistan, Bangladesh, Bhutan, India, Maldives, Nepal, Pakistan, Sri Lanka).	No studies from outside South Asian region will be included.
Time frame will be from 2005 to January 2020.	
Literatures published on English	Literatures of other languages will be excluded.
Outpatients of hospitals, clinics, physician chambers, pharmacy including self-medicated adult patients of all ages.	In-patients, Emergency patients, ICU patients.

### 3.2 Information sources

MEDLINE, Cochrane library, CINAHL Plus (EBSCO), and Web of Science will be searched using keywords including “antibiotics, antimicrobial resistance, respiratory pathogens, acute respiratory infections, South-Asia” (Table 2). In order to obtain any unclear and unreported information, authors will be contacted.

**Table 2:** Library search strategy for MEDLINE.

#	Search terms and combinations
1	“antimicrobial resistance”
2	“antimicrobial susceptibility^∗^”
3	1 OR 2
5	pneumonia
6	bronchitis
7	tonsillitis
8	tonsillitis
9	cough^∗^
10	runny nose^∗^
11	sinusitis
12	sore throat
13	breathlessness
14	asthma^∗^
15	“Upper respiratory tract infection”
16	pharyngitis
17	5 OR 6 OR 7 OR 8 OR 9 OR 10 OR 11 OR 12 OR 13 OR 14 OR 15 OR 16
16	rational use^∗^
17	dispense^∗^
18	prescribe^∗^
19	“over the counter”
20	”community^∗^ pharmacist^∗^”
21	misuse^∗^
22	“self medication^∗^”
23	“patient^∗^ compliance^∗^”
24	16 OR 17 OR 18 OR 19 OR 20 OR 21 OR 22 OR 23
25	(india OR bangladesh OR srilanka OR nepal OR bhutan OR pakistan OR maldives OR afghanistan OR “south asia”)
26	4 AND 15 AND 24 AND 25

### 3.3 Participants

We will include studies on outpatients of all ages with ARIs where data on antibiotic use is available. We will exclude inpatients, as they are more likely to receive antibiotics after a proper diagnosis. Whereas outpatients are often and less rationally prescribed antibiotics without an appropriate diagnosis as previously reported.^[14]^ We will also include participants prescribed by the pharmacy, quacks, physician's chamber, nurses, and self-medicated.

### 3.4 Interventions & Comparator

The systematic review will consider studies where participants receive antibiotics for an episode of ARI. There is no comparator, as we will not review any effectiveness.

### 3.5 Outcomes

In all populations, the primary outcomes are the proportions of patients receiving antibiotics for an episodes of acute respiratory tract infection and the resistance of antibiotics against common respiratory pathogens.

### 3.6 Search strategy

To improve the quality of reporting, we will search, collect, and evaluate the literature on antibiotic resistance and prescribing patterns for acute respiratory tract infections in south Asia based on Preferred Reporting Items for Systematic Reviews and Meta-Analysis Protocol (PRISMA-P) checklist.^[20]^ We will identify community and hospital-based studies by searching four databases using a comprehensive literature search strategy. A database-specific contextualized search strategy will be used for all four databases combining the search terms using Boolean operators. References and citations will be tracked using selective snowballing to manually extract any relevant study missed by the electronic search.

### 3.7 Study selection

We will select the study in 2 stages. The first stage will consist of studies acquired after the search strategy is deployed; these studies will be uploaded in EndNote X9 (Thomas Reuters, New York, NY) and duplicates will be identified and removed. After checking and removing the duplicates, 2 reviewers from the review team (SA and MZH) will independently screen titles and abstracts according to the inclusion and exclusion criteria.

In the second stage of screening, remaining studies will be retrieved in full and full texts of all included articles will then be screened by 2 reviewers against the inclusion and exclusion criteria using the prioritization and sequential exclusion technique. The reasons for the exclusions of the studies will be reported in the final report. In the case of disagreement between reviewers, a third reviewer will be asked to resolve any discrepancies. Results of screening and selection of studies will be presented using the PRISMA flow diagram and reported in the final systematic review.

### 3.8 Data extraction

Reviewers will extract the following information: country of publication, study setting, design, sample size, patient characteristics (age, sex, demographic, etc), the proportion of patients receiving antibiotics, commonly used antibiotics and proportion of antibiotics used according to different categories, distribution of respiratory pathogens, sensitivity and resistance pattern of the pathogens. The third reviewer will review and randomly check the data extraction process to resolve any discrepancies. The data will be collected in Microsoft Excel for analysis.

### 3.9 Data synthesis

A narrative synthesis of the findings will be conducted from the included studies, structured around the prevalence of antibiotic exposure, population characteristics, and associations between antibiotic use and demographic characteristics. We anticipate insufficient data for a meta-analysis due to the narrow range of outcomes measured. However, studies that have used the same types of exposure, and outcome measures will be pooled using a random-effect model meta-analysis. Standardized mean differences will be reported for continuous outcomes and risk ratios for dichotomous outcomes. We will calculate the 95% confidence intervals and *P* values for each outcome. We will also assess evidence of publication bias using the funnel plot and Egger test. However, publication bias will be assessed by funnel plot if there are at least 10 studies included.^[21]^

### 3.10 Quality assessment of included studies

Quality of included studies will be assessed using The Joanna Briggs Institute (JBI) Critical Appraisal Tools for all individual studies for assessing quality and susceptibility to bias in observational studies in epidemiology as appropriate to each individual study design. These include appropriate source population and inclusion and methods outlined to deal with any design-specific issues, appropriate design, and/or analytical methods; appropriate use of statistics for primary analysis of effect; and declarations of conflict of interest or identification of funding sources. Considering the scope of the review, there is possibility of not getting any experimental studies. However, if included, for the assessment of the risk of bias of included RCTs, JBI Critical Appraisal tool will be used focusing on the specific points of allocation concealment, random sequence generation, blinding at the level of participants, implementer, and outcome assessor and any other potential source of bias such as contamination. Analytical studies with case–control and cohort design will be assessed using the designated JBI quality assessment tools for case–control studies and cohort studies respectively. We will use the JBI quality assessment checklist for prevalence studies to assess the quality of cross-sectional studies. Two authors will assess the risk of bias independently using the above-mentioned tools. Any potential conflicts will be resolved by a third reviewer.

### 3.11 Analysis of subgroups or subsets

We will perform subgroup analyses by types of pathogens and antibiotics if the necessary data are available.

### 3.12 Dissemination plan

A manuscript incorporating the findings of the review will be developed and submitted for publication.

## 4 Discussion

The findings of this systematic review will contribute to future research and policy including the development of revised ARI guidelines for clinicians for the treatment of ARI based on current AMR data on the respiratory pathogen. The proportion of patients receiving antibiotic therapy for ARI, the proportion of different types of antibiotics received, and resistance against respiratory pathogen might help to promote the rational use of antibiotics in ARI. This systematic review will include literature published only in English. This will be a limitation to this review as there might be few non-English research papers retrieved from the search. However, to our knowledge, no scientific articles are published in South Asian countries’ local language. This systematic review does not require any ethical approval, as all the data will be collected from secondary sources and published literatures. All the findings will be summarized in a single manuscript and disseminated through peer-reviewed publications.

## Acknowledgment

MZH and FC acknowledge the salary support from ICDDRB through a grant from the US Centers for Disease Control and Prevention. SA acknowledge the salary support from the Graduate School of the University of North Carolina at Greensboro.

## Author contributions

MZH and SA conceived the idea and drafted the protocol. KMSUR and MABC revised the protocol. SA, MZH, FC, SDM critically reviewed it. SBD, SA, and KMSUR refined the search parameter following the consultation with other authors. All authors read and approved the final protocol manuscript. MZH is the guarantor of the review.

**Conceptualization:** Md. Zakiul Hassan and Saif Al Amin

**Formal analysis:** Saif Al Amin, Md Zakiul Hassan, KM Saif-Ur-Rahman, Fahmida Chowdhury, Muhammad Abdul, Baker Chowdhury

**Methodology:** Saif Al Amin, Md. Zakiul Hassan, KM Saif-Ur-Rahman, Fahmida Chowdhury, Sara B. Donevant, Sharon D. Morrison

**Software:** Saif Al Amin

**Supervision:** Md. Zakiul Hassan, KM Saif-Ur-Rahman, Sharon D. Morrison, Fahmida Chowdhury.

**Validation:** Saif Al Amin, KM Saif-Ur-Rahman, Sara B. Donevant.

**Writing – original draft:** Saif Al Amin and Md. Zakiul Hassan

**Writing – review & editing:** Fahmida Chowdhury, KM Saif-Ur-Rahman, Muhammad Abdul Baker Chowdhury, Sara B. Donevant, Sharon D. Morrison

## Abbreviations

Abbreviations: ARI = acute respiratory infection, AMR = antimicrobial resistance, URTI = upper respiratory tract infection.
